# Personalized nutrition therapy in critical care: 10 expert recommendations

**DOI:** 10.1186/s13054-023-04539-x

**Published:** 2023-07-04

**Authors:** Paul E. Wischmeyer, Danielle E. Bear, Mette M. Berger, Elisabeth De Waele, Jan Gunst, Stephen A. McClave, Carla M. Prado, Zudin Puthucheary, Emma J. Ridley, Greet Van den Berghe, Arthur R. H. van Zanten

**Affiliations:** 1 Department of Anesthesiology and Surgery, Duke University School of Medicine, Box 3094 Mail # 41, 2301 Erwin Road, 5692 HAFS, Durham, NC USA; 2 Departments of Nutrition and Dietetics and Critical Care, Guy’s and St Thomas’ NHS Foundation Trust, London, UK; 3 Faculty of Biology & Medicine, Lausanne University, Lausanne, Switzerland; 4 Department of Clinical Nutrition, Universitair Ziekenhuis Brussel, Brussels, Belgium; 5 Vrije Universiteit Brussel, Brussels, Belgium; 6 Clinical Division and Laboratory of Intensive Care Medicine, Department of Cellular and Molecular Medicine, KU Leuven, Louvain, Belgium; 7 Department of Medicine, University of Louisville School of Medicine, Louisville, KY USA; 8 Department of Agricultural, Food and Nutritional Science, University of Alberta, Edmonton, AB Canada; 9 William Harvey Research Institute, Queen Mary University of London, London, UK; 10 Royal London Hospital, Barts Health NHS Trust, London, UK; 11 Australian and New Zealand Intensive Care Research Centre, School of Public Health and Preventive Medicine, Monash University, Level 3, 553 St Kilda Rd, Melbourne, VIC 3004 Australia; 12 Dietetics and Nutrition, Alfred Hospital, 55 Commercial Rd, Melbourne, VIC 3004 Australia; 13 Department of Intensive Care, Gelderse Vallei Hospital, Wageningen University & Research, Ede, The Netherlands

**Keywords:** Critical illness, Indirect calorimetry, Protein, Parenteral nutrition, Enteral nutrition, Micronutrients, ICU, TPN, Nutrition, Testosterone, Muscle, Body composition

## Abstract

Personalization of ICU nutrition is essential to future of critical care. Recommendations from American/European guidelines and practice suggestions incorporating recent literature are presented. Low-dose enteral nutrition (EN) or parenteral nutrition (PN) can be started within 48 h of admission. While EN is preferred route of delivery, new data highlight PN can be given safely without increased risk; thus, when early EN is not feasible, provision of isocaloric PN is effective and results in similar outcomes. Indirect calorimetry (IC) measurement of energy expenditure (EE) is recommended by both European/American guidelines after stabilization post-ICU admission. Below-measured EE (~ 70%) targets should be used during early phase and increased to match EE later in stay. Low-dose protein delivery can be used early (~ D1-2) (< 0.8 g/kg/d) and progressed to ≥ 1.2 g/kg/d as patients stabilize, with consideration of avoiding higher protein in unstable patients and in acute kidney injury not on CRRT. Intermittent-feeding schedules hold promise for further research. Clinicians must be aware of delivered energy/protein and what percentage of targets delivered nutrition represents. Computerized nutrition monitoring systems/platforms have become widely available. In patients at risk of micronutrient/vitamin losses (i.e., CRRT), evaluation of micronutrient levels should be considered post-ICU days 5–7 with repletion of deficiencies where indicated. In future, we hope use of muscle monitors such as ultrasound, CT scan, and/or BIA will be utilized to assess nutrition risk and monitor response to nutrition. Use of specialized anabolic nutrients such as HMB, creatine, and leucine to improve strength/muscle mass is promising in other populations and deserves future study. In post-ICU setting, continued use of IC measurement and other muscle measures should be considered to guide nutrition. Research on using rehabilitation interventions such as cardiopulmonary exercise testing (CPET) to guide post-ICU exercise/rehabilitation prescription and using anabolic agents such as testosterone/oxandrolone to promote post-ICU recovery is needed.

## Introduction

The wisdom of Lord Kelvin and Galileo is essential in ICU where our ability to measure human physiology and its response to illness, as well as to our interventions is critical [1]. Kelvin's statement was actually, *“When you can measure what you are speaking about, express it in numbers, you know something about it; but when you cannot measure numbers, your knowledge is meager and unsatisfactory; it may be beginning of knowledge, but you have scarcely…advanced to stage of science*[1]*.”* Current ICU nutrition therapy has remained at “*beginning of knowledge”* in personalizing care*.* We are limited in objectively measuring ICU patient's nutrition needs and metabolic/clinical responses to nutritional interventions. This often leaves us feeling our understanding of ICU nutrition is “*meager and unsatisfactory.”*

**Fig. 1:**
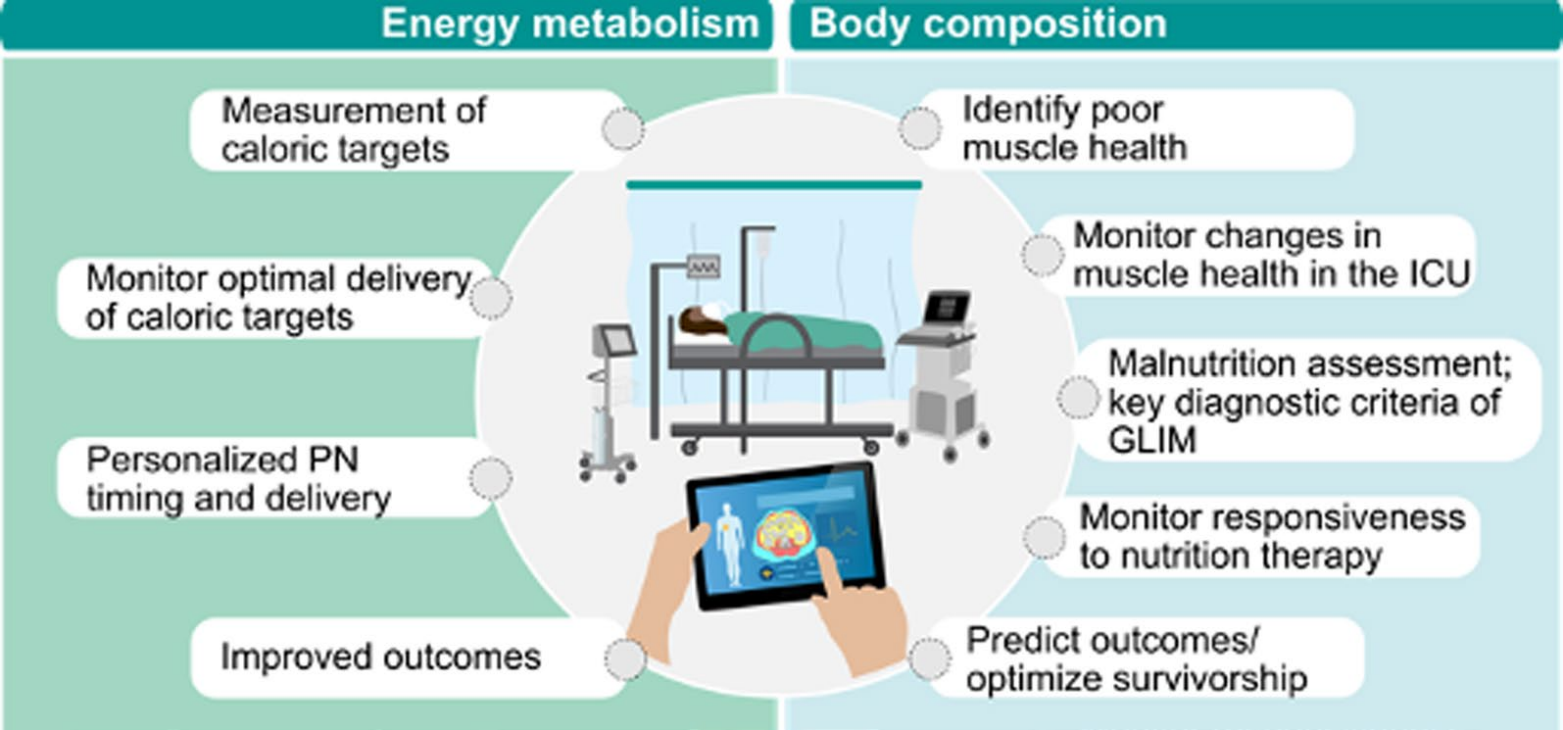
Future goals for personalization of nutrition in critical care via assessment of energy metabolism and body composition assessment. GLIM—Global Leadership Initiative on Malnutrition, PN—parenteral nutrition

A driver of lack of emphasis on ICU nutrition is lack of objective data to guide nutrition and measure metabolic, muscle and physical function responses to nutrition strategies. To emphasize, *ICU physicians would never deliver vasopressors without accurate blood pressure measurements from an arterial line/cuff; thus, we believe ICU community has not embraced focus on nutrition being equally important to other care due to lack of ability to objectively provide “measures” to guide care*[1]*.* Ideally, nutrition should be individualized with “ready-to-feed” indicators and markers indicating when energy delivery is advanced and protein incorporated into lean mass (LM). Further, we must determine when energy intake is adequate while minimizing over/underfeeding. Thus, we must evolve current/future devices for measurement of energy needs and body composition to meet goals in Fig. 1.

This manuscript describes current progress in ICU nutrition and highlights where research is needed on ten personalized nutrition questions. Current guideline recommendations from ESPEN/ASPEN and practice suggestions incorporating recent literature summarized in Table 1**.**

**Table 1:** Personalized Nutrition in Critical Care: Practical Practice Recommendations, Current Guideline Recommendations, and Future Research Priorities

	Question	Suggested expert answer per recent data:	Most recent guideline recommendations	Research priorities	Examples of key ongoing trials
1	Personalizing when to start nutrition?	While EN is the preferred route of early nutrition delivery, new data highlight isocaloric doses of PN can be given safely without increased risk vs. EN, such that when EN is not feasible, provision of early PN over the short term is effective and results in similar outcomes. Key message is early high-dose feeding should be avoided until the patient is stabilized early in ICU stay Trophic EN may prove to exert non-nutritional benefits on microbiome, gut barrier function, vagal nerve signaling, and mesenteric lymph toxicity	ASPEN 2021—Although EN is preferred, either PN or EN is acceptable as the primary early feeding modality in the first week of critical illness. EN and PN may be considered equivalent in terms of risk and outcome benefits ESPEN 2019—If oral intake is not possible, early EN (within 48 h) in adult ICU patients should be performed/initiated rather than delaying EN ASPEN/SCCM 2016—We recommend nutrition care in the form of early EN be initiated within 24–48 h in the ICU patient unable to maintain volitional intake	Research/development of new “ready-to-feed” indicators or markers to assist understanding when is most optimal to initiate nutrition delivery Research on optimal timing to start nutrition based on non-nutritional benefits of EN, even trophic EN, on microbiome/dysbiosis, gut barrier function, vagal nerve signaling, and mesenteric lymph toxicity	—SendHOME Trial: Personalized Targeted Nutrition Via StructurEd Nutrition Delivery Pathway to Improve Recovery of Physical Function in Trauma (RCT)—(starting 7/2023)—Duke University—Funded by Department of Defense
2	Personalizing how much to feed: Role of Indirect Calorimetry?	IC measurement is gold standard measurement of EE IC measurements of EE are recommended by current ESPEN/ASPEN guidelines after stabilization of patient post-ICU admission (after day 2–3) to attempt to prevent over-/underfeeding. The evolution of IC technology has made utilization of IC targets more practical for centers worldwide to consider in clinical practice In acutely ill patients, early endogenous glucose production cannot be suppressed by providing nutrition. In such case, matching the energy target with measured EE could lead to overfeeding In well-nourished mechanically ventilated patients admitted to ICU on high doses of vasopressors (doses of > 0.2 ug/kg/min norepinephrine), lower energy doses (5–10 kcal/kg/d and < 0.4 g/kg/d of protein) can be considered to be delivered until day 7 or until patient is extubated and weaning off of vasopressors Predictive equations need to be used in early acute phase (up to day 3) and can be used patients when IC cannot be utilized If IC is used: Delivery below EE target (~ 70%) during early phase (day 3 to ~ day 7) is suggested—to be increased gradually to match EE later in stay as patient recovers. As patient recovers and starts physical therapy, the addition of an activity factor to REE may be needed and can be considered In general, predictive equations are inaccurate and often lead to over and underfeeding. However, in patients not able to have energy needs measured via IC or where IC is not available—predictive equations need to be used (i.e., Penn State in ventilated patients)	ASPEN 2021—We suggest feeding between 12–25 kcal/kg (i.e., the range of mean energy intakes examined) in the first 7–10 days of ICU stay ESPEN 2019—To avoid overfeeding, early full EN and PN shall not be used in critically ill patients but shall be prescribed within three to seven days —EE should be measured by IC where available —If IC is used, isocaloric nutrition rather than hypocaloric nutrition can be progressively implemented after the early phase of acute illness —Hypocaloric nutrition (not exceeding 70% of EE) should be administered in the early phase of acute illness —After day 3, energy delivery can be increased up to 80–100% of measured EE —If predictive equations are used to estimate the energy need, hypocaloric nutrition (below 70% estimated needs) should be preferred over isocaloric nutrition for first week of ICU stay ASPEN/SCCM 2016—Suggest IC be used to determine energy requirements in absence of variables that affect accuracy of measurement	Trials utilizing IC-guided nutrition delivery to generate evidence to better define energy needs in different populations and demonstrate further evidence of clinical outcome benefits of personalization of nutrition delivery via IC, especially after acute phase of critical illness and in post-ICU recovery period Study of utility of RQ measurements to determine response to nutrition interventions and recovery from ICU	—SendHOMETrial (RCT)—(starting 7/2023)—Duke University—Funded by Department of Defense
3	Personalizing Protein Delivery: How much and timing?	Low-dose (e.g., ≤ 0.8 g/kg/day) protein delivery can be considered during the early acute phase (up to day 3)—to be progressively increased > 1.2 g/kg/d later. Higher doses of protein should be avoided in unstable patients (i.e., in shock on higher doses of vasopressors) In well-nourished mechanically ventilated patients admitted to ICU in shock on high doses of vasopressors (doses of norepi. ≥ 0.2 ug/kg/min) < 0.4 g/kg/d of protein can be considered to be delivered until patient is stabilized and weaning off of vasopressors Higher doses of protein (> 1.2 g/kg/d) should potentially be avoided in patients presenting with acute kidney injury (stage 1–3) (not or CRRT) and high SOFA score (≥ 9)	ASPEN 2021—Given limited new data continue to utilize 2016 ASPEN/SCCM guideline suggestion for 1.2–2.0 g/kg/day ESPEN 2019—During critical illness, 1.3 g/kg protein per day can be delivered progressively	Studies of role of bedside techniques such as BIA, muscle ultrasound and new validated biomarkers can facilitate nutritional protein therapy individualization. (i.e., use of BIA or other muscle measures to base protein delivery on lean mass as opposed to total body weight) Development of markers of protein utilization and incorporation into muscle and progression/resolution of anabolic resistance Urea/creatinine to monitor catabolism and intervention response	—NEXIS trial: Nutrition and Exercise in Critical Illness Trial (NEXIS Trial): a protocol of a multicentered, randomized controlled trial of combined cycle ergometry and amino acid supplementation commenced early during critical illness (multicenter RCT) (Ongoing)-NIH funded NCT#: 03021902 Protocol Publication: https://bmjopen.bmj.com/content/9/7/e027893 —TARGET Protein: The effect of augmented administration of enteral protein to critically ill adults on clinical outcomes: A cluster randomized, cross-sectional double crossover, registry-embedded, pragmatic clinical trial (Cluster randomized RCT) (Ongoing 3000/4000 enrolled) Link: https://www.australianclinicaltrials.gov.au/anzctr/trial/ACTRN12621001484831 —PRotEin Provision in Critical IllneSs (PRECISe) (Randomized multicenter trial) NCT# 04633421
4	Personalization of PN Timing and Delivery?	If EN is not able to be started early PN may be started safely w/o increased risk and results in similar outcomes. Early high-dose PN feeding should be avoided until the patient is stabilized during ICU stay EE measurements targets should be made with IC which may assist with determining PN energy dose (when possible). Below EE (~ 70%) should be considered during the early phase (4–7 days) Energy delivery to be increased to match EE later in stay	ASPEN 2021—In adult critically ill patients, we recommend that either PN or EN is acceptable as the primary early feeding modality in the first week of critical illness. EN and PN may be considered equivalent in terms of risk and outcome benefits ESPEN 2019—In case of contraindications to oral and EN, PN should be implemented within three to seven days Early and progressive PN can be provided instead of no nutrition in case of contraindications for EN in severely malnourished patients	Studies on role of IC guidance to personalize PN and SPN delivery and potentially improve clinical and long-term functional outcomes in ICU patients Studies of role of early PN and SPN use on post-ICU functional outcomes and MM/function	—INTENT Trial: Intensive Nutrition Therapy comparEd to usual care iN criTically ill adults (INTENT): a phase II randomized controlled trial (Phase II multicenter RCT) (completed)—ANZICS group: NCT#: 03292237 Protocol Publication: https://bmjopen.bmj.com/content/12/3/e050153 —SendHome Trial (RCT)—(starting 7/2023)—Duke University—Funded by Department of Defense
5	Personalization of Feeding and Fasting Periods?	Recent evidence suggests that the lack of early full feeding in large RCTs may be explained by the delivery mode of artificial nutrition. Switching from continuous nutrition to intermittent nutrition, hence alternating feeding and fasting intervals may be superior, although confirmatory RCT evidence is needed In case of GI dysfunction impairing the tolerance of full-volume isocaloric EN, fluid restriction or transitioning to oral nutrition using an intermittent-feeding schedule (e.g., overnight) can be considered safely given recent data (ref. 69–70)	ESPEN 2019—Continuous rather than bolus EN should be used ASPEN/SCCM 2016—Based on expert consensus, we suggest for high-risk patients or those shown to be intolerant to bolus gastric EN, EN delivery be switched to continuous infusion	Further studies should investigate how efficacy and safety of intermittent feeding/fasting can be monitored. The ideal feeding/fasting regimen (if any) also remains to be determined Studies of Ketogenic substrates such as ketones in ICU Outcomes assessing muscle mass/function and post-ICU functional and quality of life outcomes should be key endpoints in these studies	—Alternative Substrates in the Critically Ill Subject (Randomized Controlled Pilot trial) (ASICS) (Recruitment completed) NCT#: NCT04101071 —Effect of Continuous Versus Cyclic Daytime Enteral Nutrition on Circadian Rhythms in Critical Illness (CIRCLES) (Randomized controlled trial) (Not yet recruiting) NCT#: 05795881
6	Personalizing Monitoring of Nutrition Delivery?	It is critical ICU clinicians are aware on a daily basis of delivered energy/protein and what percentage of personalized goal nutrition targets this delivered amount represents New computerized nutrition monitoring systems and full-automated ICU nutrition platforms are now widely available	No specific recommendations	Studies of integrated nutrition delivery platforms and nutrition delivery monitors to assess effect on clinical and functional outcomes	
7	Personalizing Monitoring and Repletion of Micronutrient Deficiencies?	The majority of micronutrient analysis should be initiated after 5–7 days in the ICU, i.e., in patients with risk factors for nutrient losses as follows: patients with active losses of biological fluids, especially continuous renal replacement therapy (CRRT) CRRT is now understood to lead to significant losses and low plasma levels in multiple micronutrients and water-soluble vitamins in ~ 90% of patients within 5–7 days of CRRT initiation. Additionally, intestinal losses, major drains, and major burns lead to MN deficiencies If repletion initiated, monitoring results is required within a ~ week. CRP should be utilized when trace element levels assessed	ESPEN Micronutrient Guideline: Adequate amounts of all essential trace elements and vitamins shall be supplied to all patients receiving medical nutrition from the beginning of the period of nutritional support C-reactive protein should be determined at the same time as any micronutrient analysis to assist with interpretation	Trials of role of routine micronutrient supplements to prevent micronutrient deficiencies in patients at high risk of nutrient deficiencies (especially CRRT patients). Focus on clinical and long-term functional outcomes	—Lessening Organ Dysfunction With Vitamin C (LOVIT) Trial (Randomized MultiCenter Trial) (Enrollment Completed): NCT#: 03680274 Protocol Paper: Doi: 10.2196/36261
8	Personalizing Monitoring Of Catabolism And Muscle Mass And Effect Of Nutrition On Muscle Recovery?	Routine use of modalities such as muscle ultrasound, CT scan, and/or BIA monitoring to assess nutrition risk, effect of/response to nutrition delivery are widely used in ICU research and may become a future standard of care in clinical ICU care and clinical /translational ICU nutrition research to personalize nutrition care	GLIM Criteria 2018—Muscle mass is now a key malnutrition diagnostic criterion. Beginning to be required by insurers in USA for validation of malnutrition diagnosis	Trials examining modalities such as muscle ultrasound, CT scan, and/or BIA monitoring to assess nutrition risk, effect of/response to nutrition delivery, and accurate determination of nutrition needs (i.e., BIA or other measures of lean mass to more accurately determine protein dose) Urea/creatinine to monitor catabolism and intervention response	
9	Personalizing Use of Specialized Anabolic Nutrients?	HMB, creatine, and leucine may be promising at improving strength, MM, and lean mass given data in other clinical populations. More data in ICU populations are needed	No specific recommendations	Further studies of HMB, creatine, and leucine on muscle strength, MM, and lean mass given data in ICU populations are needed. Trials also should examine long-term functional outcomes Urea/creatinine to monitor catabolism and intervention response	A study to investigate the effect of HMB on skeletal muscle wasting in early critical illness (HMB-ICU). NCT# 03464708
10	Personalizing Post-ICU Physical Function Recovery via Combination of Personalized Nutrition, Exercise, and Anabolic Pharmacologic Agents?	Post-ICU Personalized Nutrition: Continued use of indirect calorimetry, MM and energy states analysis can be considered post-ICU to personalize nutrition delivery in critical post-ICU recovery period. This is an area of significant research need Post-ICU Personalized Exercise: The potential to guide and personalize post-ICU exercise with CPET testing and VO2 peak step testing is promising and is undergoing current research in clinical trials and deserves additional research effort Post-ICU Anabolic Pharmacologic Agents: Data for oral oxandrolone are positive for clinical and functional outcome improvements in burns. A vast majority of ICU patients are severely testosterone deficient. More research in broader ICU populations is needed to determine who will optimally benefit from testosterone/oxandrolone treatment	ESPEN 2019—Physical activity may improve the beneficial effects of nutritional therapy ASPEN/SCCM 2016—Based on expert consensus, we suggest that chronically critically ill patients (defined as those with persistent organ dysfunction requiring ICU LOS > 21.days) be managed with aggressive high-protein EN therapy and, when feasible, that a resistance exercise program be used	Studies examining use of IC, MM, and EE analysis in post-ICU setting to personalize nutrition delivery needed Trials of personalized post-ICU exercise with CPET testing and VO2 peak step testing as well as monitoring of exercise at home via mobile technologies urgently needed Studies of oxandrolone/testosterone agents as part of nutrition/exercise intervention in non-burn ICU patients and post-ICU patients needed. Endpoints should include long-term post-ICU functional and MM/function endpoints (i.e., 6 min walk test, muscle strength, QOL)	—REmotely Monitored, Mobile Health Supported Multidomain Rehabilitation Program With High Intensity Interval Training for COVID-19 (REMM-HIIT-CoV) (Multicenter RCT) (NIH Funded) (Currently enrolling) NCT#: 05218083 —NEXIS trial (multicenter RCT) (Ongoing)-NIH funded NCT#: 03021902 Protocol Publication: https://bmjopen.bmj.com/content/9/7/e027893 —Personalized Nutrition Delivery to Improve Resilience in Older Adult Trauma Patients (SeND Home) (Randomized trial) (enrolling) NCT#: 05544162 —Optimised Nutritional Therapy and Early Physiotherapy in Long Term ICU Patients (NutriPhyT Trial) (NutriPhyT) (Randomized clinical trial) (Enrolling) NCT# NCT05865314

EE: Energy expenditure; IC—indirect calorimetry; Micronutrients = trace elements + vitamins, MM—muscle mass

### Question 1: When do we start nutrition?: Personalization of initiating nutrition in ICU

Societal guidelines emphasize initiation of early enteral nutrition (EN) [2–6] with rationale that changes in gut barrier are seen within 24 h and include evidence of intestinal ischemia, increased permeability, bacterial translocation, and dysbiosis [7–11]. Current literature supports early EN(EEN) may attenuate changes via range of “non-nutritional benefits” and improve outcomes compared to delayed EN(DEN) [6]. Recent meta-analyses show EEN versus DEN associates with fewer complications [12], infectious morbidity [2, 4, 6], and ICU/hospital length of stay(LOS) [2, 13]. In two meta-analyses, EEN associates with significant reduction in mortality [4, 12], a benefit not confirmed by two other meta-analyses [2]. EEN benefit is supported in COVID-19 in national database study showing reduced ventilator time and reduced ICU/hospital LOS when COVID-19 ICU patients are fed within three days of admission [14].

EEN timing in guidelines ranges from first 24 h [5] or 24–48 h [2, 6] of ICU admission. Delay or slowing EN advancement is suggested in GI bleeding, mesenteric ischemia, GI intolerance (i.e., GRV > 500 ml), risk of aspiration, intestinal obstruction, abdominal compartment syndrome, risk of refeeding syndrome (or phosphate < 0.65 mmol/L) or unresuscitated hemodynamic instability on vasopressors [2, 5]. No delay is suggested on vasopressors (norepinephrine < 0.3 ug/kg/min) who are adequately resuscitated (i.e., normalized lactate) [8], open abdomen, neuromuscular blockade, therapeutic hypothermia, ECMO, or prone positioning [6].

In patients with EEN contraindications, parenteral nutrition (PN) personalization is described in Question 4. However, higher doses of either EN/PN should be avoided on high doses of vasopressors (norepinephrine ≥ 0.3 ug/kg/min) prior to reasonable vasopressor weaning. This is reinforced by Nutrirea-3 trial showing increased ICU LOS by one day in intubated patients on vasopressors (admit norepinephrine:0.5 ug/kg/m) in low-dose nutrition group (6 kcal/kg/day//0·2–0·4 g/kg/day protein) *vs* full-nutrition (25 kcal/kg/day//1·0–1·3 g/kg/day protein) [15]. No difference in mortality/infection was observed [15]. *Key message is early high-dose feeding should be avoided until patient is stabilized early in ICU stay*.

### Question 2: How much energy to feed? Personalization of energy targets via indirect calorimetry

Key role of objective energy expenditure (EE) measurement in ICU has recently been described and is possible in routine practice as indirect calorimetry (IC) technology has evolved [16]. Further, respiratory quotient (RQ) provides information on underfeeding (RQ < 0.7), technical problems (leaks), or overfeeding (RQ > 1.0). A key retrospective study on ~ 1200 ICU patients associated delivering ~ 70% of resting IC-measured EE (REE) with improved ICU survival [17]. Both underfeeding and overfeeding beyond 80% of IC targets associated with increased mortality in acute ICU phase [17]. Its well-known predictive equations (PE) fail to predict EE in ICU with correlation factors of 0.24–0.73 for 12 equations [18, 19]. In recent data, IC-measured metabolic rate of COVID-19 patients deviated significantly from values obtained by all common PE [20, 21].

**Fig. 2:**
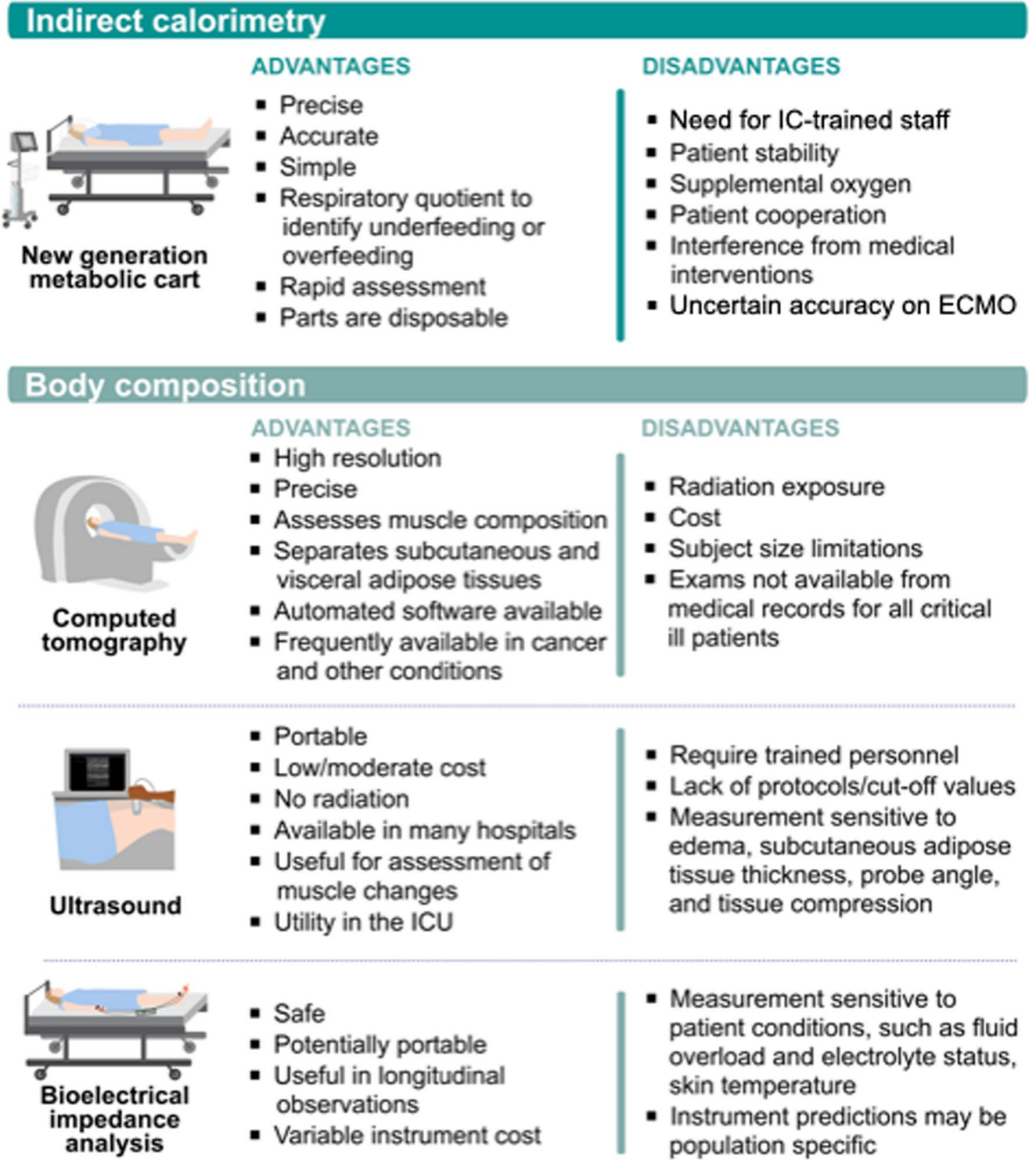
Advantages and disadvantages of emerging indirect calorimetry devices and body composition techniques in critical care

Thus, although IC-guided nutritional targets and EE measures may be essential to future ICU nutrition personalization, IC data must be used judiciously. Measurements should not be performed too early in ICU before patients are adequately resuscitated (~ post-ICU day 3). Further, all recent ICU nutrition guidelines suggest energy delivery start at ~ 10–15 kcal/kg or < 70% of measured-IC REE whether EN/PN is used and advance as patient stabilizes. If additional insult occurs (i.e., new sepsis), reduction to 10–15 kcal/kg/ < 70% IC-measured EE may be needed, further research will need to clarify this. European/American guidelines both advocate IC to measure EE [2, 22]. Recent meta-analysis data showed no effect of IC-directed nutritional therapy on mortality, but IC-directed care may prolong mechanical ventilation [23]. Subsequently, two recent meta-analyses demonstrate potential clinical benefit of IC-guided nutrition. Both showed significantly reduced ICU mortality with IC-guided energy targets [24–26]. Worldwide availability/evolution of IC technology has made it increasingly accurate and simple to obtain IC-measures and more practical for centers worldwide to consider adding IC to practice [16, 27, 28]. Newer IC devices provide accurate REE measures in ~ 5–10 min, with time required to obtain EE measurements significantly shorter with new-IC devices (*p* < 0.001) versus previous devices (including: Deltatrac®/Quark RMR®/V-max®) [29]. Further, accurate readings are now possible, up to 70% FiO_2_, and data show that REE can be made with IC on CRRT [30]. In centers with trained staff (including dietitians), IC-measured EE in appropriate patients can help prevent over-/underfeeding, common in ICU [17]. This can bring greater focus/objective data to nutrition therapy and its crucial role in ICU and post-ICU setting, when nutrition may play a more significant role in clinical/functional recovery [31]. Given new technology, we must focus on larger trials to generate evidence to define energy needs in different populations and demonstrate potential further evidence of benefits of nutrition personalization via IC [16, 27]. (See Fig. 2: advantages/disadvantages of new-ICs).

### Question 3: How much protein to deliver? Personalizing protein dose and timing

Marked muscle mass (MM) loss is seen during ICU stay [32]. Optimal delivery of amino acids (AA) is essential for protein homeostasis and counteracts catabolism in healthy subjects [33]. In ICU, higher protein delivery associates with improved outcomes and reduced MM loss in observational studies [17, 34, 35]. International guidelines recommend advancing protein to 1.3–2.0 g/kg/day [2, 5]. However, recent meta-analysis showed high protein was not associated with improvement in clinical or patient-centered outcomes [36], and recent EFFORT-Protein trial demonstrated no benefit of high doses (> 2.2 g/kg/d) [37]. It is vital to understand enhanced AA provision may not increase muscle protein synthesis (MPS) in acute phase. A recent study showed ICU patients have 60% less MPS vs. healthy subjects, despite normal gut protein absorption [38]. In ICU, anabolic response may be blunted due to variations in anabolic resistance (lower effect of protein and exercise on MPS), immobilization, insulin resistance, inflammation, decreased satellite cell numbers, and low muscle ATP muscle [32, 33]. Some studies investigating higher protein show adverse effects such as enhanced muscle wasting, autophagy inhibition, increased ureagenesis, prolonged organ failure, and LOS [32, 39, 40]. Recently, it was suggested protein intake timing plays a role*,* as early high protein (> 0.8 g/kg/d) associated with higher mortality; however, higher protein during days 4–7 (> 1.2 g/kg/d) associated with improved survival [41]. Moreover, even when protein preserves MM, this does not always translate into improved muscle function/strength [42]. Subgroup analysis of EFFORT-Protein trial and in REDOX suggests worse outcomes between protein dose and patients with AKI (stage 1–3) not receiving baseline CRRT and high admit SOFA score(≥ 9) [37, 43]. Therefore, optimal timing/protein dosing based on individual patient characteristics during ICU is key. But how?

First, total body weight (TBW) is typically used to calculate protein dose. However, protein calculations may preferably be based on lean mass (LM). In sarcopenic obesity, TBW targets may induce protein overdosing; however, in non-sarcopenic obesity, underdosing may occur. Bioimpedance Assessment (BIA) can be used as bedside tool to estimate LM [44]. Alternatives are muscle ultrasound and CT scans. Otherwise, Gallagher equation to estimate LM may be applied, although estimations are based on population averages and may misinterpret body composition [45]. As most studies have not included body composition when studying effect of protein, significant variations in protein dose per LM may be expected, challenging available evidence. Second, biomarkers on muscle breakdown, autophagy, inflammation, and insulin resistance may identify patients benefitting from higher protein. However, we lack specific validated biomarkers and evidence they improve outcome. Third, nitrogen (N) balance, reflecting equilibrium between protein intake and losses (via urinary urea), may help. Studies suggest positive nitrogen balance achieved by providing higher protein associates with improved outcomes [46]. Conversely, in studies on high-protein delivery, enhanced ureagenesis and urinary nitrogen loss have been observed, questioning whether higher intake equals MPS. Additionally, limitations are identified for N-balance studies, such as acute renal failure and urinary loss of non-urea nitrogen (e.g., ammonia, creatinine, uric acid/amino acids) [47]. Targeting protein provision to individual ICU protein requirements is challenging and still in infancy. Bedside techniques including BIA, muscle ultrasound, and new biomarkers may further facilitate individualization of protein delivery.

### Question 4: Personalization of parenteral nutrition

PN personalization may prove critical in optimizing nutrition in ICU. Trials investigating PN either alone or with EN (Supplemental PN-(SPN)) compared to standard care (lower feeding) or EN alone show variable results from SPN associated with reduced late infections [48], PN reducing mechanical ventilation time [49], PN reducing bowel ischemia events versus EEN [50], as well as PN showing increased ICU dependency [39]. Lack of personalization may be one explanation for variable results. The Heidegger trial showed benefits of early SPN on late infections utilizing IC-guided personalized energy targets [48], which may prove key to preventing over-/underfeeding with PN.

The population in whom and when to commence PN is among recent advances in ICU care and continues to require future study. The antiquated (and inaccurate) concept of “PN is harmful” for all ICU patients has been disproven. As stated, while physiologic response to PN is different from EN, and EN is still preferred, new ASPEN ICU guidelines [51] highlight when EN is not feasible, provision of PN over short-term is safe, effective, and results in similar outcomes to EN. These new guidelines and 4 large-randomized trials indicate PN is no longer associated with risk of infection [48–52]. Limited data suggest benefit of commencing EN+PN in patients at nutrition risk, but further research is needed [53, 54]. In support of this, a recent trial in major abdominal surgical patients (not all ICU) showed early SPN started at day three significantly reduced infectious complications versus waiting until day 8 for SPN [55]. A recent SPN meta-analysis showed SPN was not inferior in regards to mortality risk, hospital/ICU LOS, or ventilation days [56]. An additional recent SPN meta-analysis in ICU versus EN alone showed SPN+EN decreased risk of nosocomial infections (RR) = 0.733, *p* = 0.032) and ICU mortality (RR = 0.569, *p* = 0.030)[57].

It is critical when early PN is utilized, guidelines recommend a ramped approach to energy/protein delivery with initial doses starting at 10–15 kcal/kg or < 70% IC REE and protein starting at < 0.8 g/kg/d with advancement over first ICU week. *More research is urgently needed to understand if and how IC guidance and markers of protein utilization can be optimally utilized to personalize PN/SPN delivery and potentially improve clinical/long-term functional outcomes.*

### Question 5: Personalization of feeding and fasting periods

Recent evidence suggest lack of benefit of early full feeding in large ICU trials may be explained by method artificial nutrition provision (i.e., continuously). Indeed, alternating feeding/fasting intervals may be superior compared to continuous delivery–applied in most RCTs [58]. Potential protective mechanisms of intermittent feeding include intermittent activation of fasting response, which may promote cellular recovery via stimulation of autophagy and ketogenesis [59–61]. Further, intermittent provision of nutrients may avoid “muscle-full effect,” the observation that MPS only temporarily rises after increasing AA availability [62]. Finally, aligning feeding/fasting periods with regular diurnal pattern may attenuate disturbances in circadian rhythm, which is implicated in diseases including ICU [58, 63]. Until recently, it was unclear how long ICU patients should fast before a fasting response develops. Recently, a pilot crossover RCT revealed twelve hours of fasting-induced a metabolic fasting response in prolonged ICU patients, with increases in circulating ketones [64]. However, RCTs on intermittent versus continuous feeding are scarce and yield divergent results [65, 66]. Whereas some RCTs find more feeding intolerance with intermittent-feeding boluses, others show higher feeding intake and/or lower aspiration pneumonia by intermittent feeding [65, 66]. One RCT did not detect effect of intermittent feeding on ultrasound-assessed muscle wasting [66]. However, apart from heterogeneity in design, all RCTs were small/underpowered for clinical endpoints [65, 66]. Moreover, in all RCTs, fasting interval was restricted to 4–6 h, which may be too short to induce fasting response and its benefits [58]. Future RCTs should investigate optimal initiation of artificial feeding time, optimal dose, and ideal duration of fasting interval. Potentially, individualization of duration of fasting and energy needs during feeding interval is needed. Thus, future research should develop/validate biomarkers confirming activation of fasting response and metabolic tolerance to artificial feeding [67]. Ketones may serve as biomarkers to guide fasting interval duration.

### Question 6: How to personalize monitoring of nutrition delivery?

Numerous studies show gap between EN prescription/delivery [68]: in observational data larger gaps worsen outcome, although association may be confounded by illness severity as sicker patients may tolerate feeding poorly. Thus, it is critical clinicians are aware of delivered energy/protein on a daily basis and what percentage of personalized goal nutrition targets this delivered amount represents. New computerized information systems can or are customized to enable visualization of nutrition quantity being delivered [69]. Monitoring accurate nutrition delivery via computerized information systems increases nutrition delivery significantly [69]. Such systems now use feeding tubes equipped with captors to prevent/reduce aspiration risk in noninvasive ventilation or high-flow oxygen. A new technology even now detects presence/duration of gastro-esophageal reflux and assists in preventing aspiration real-time [70]: initial clinical trial data show an automated nutrition platform with aspiration-prevention feeding tube reduces ICU LOS (personal communication, P. Singer). See Fig. 2 for new personalized monitoring of ICU nutrition.

### Question 7: How should we personalize monitoring and repletion of micronutrient and vitamin deficiencies

*Micronutrients (MN) deficiency is quite frequent and is rarely tested for/diagnosed in ICU* [71–74]***.*** ESPEN encourages monitoring selected MNs [71, 75] in new guidelines as deficiencies can be responsible for numerous complications [76]. Recent ESPEN Guidelines on Micronutrients finally provide guidance on diagnosing/treating MNs [75]. When should MNs be monitored? Testing should be initiated after 6–7 days in ICU. Patients at risk of deficiencies are those with active depletion, especially on CRRT [73] known to lead to significant losses/low measured levels of multiple micronutrients and water-soluble vitamins in ~ 90% of patients within 5–7 days on CRRT [73, 74]. Additionally, intestinal losses, major drains, and major burns [76] lead to MN deficiencies. Inflammation, generally present in ICU, complicates interpretation of results [77]: in CRP > 40 mg/l, some MNs will be below references values not necessarily reflecting deficiency, with exception of copper, which increases with inflammation. Values 20% below laboratory's reference value should raise concern for MN's status and trigger repletion with PN multi-trace element/vitamins [78] or administration of repletion doses if lower. When repletion is initiated, monitoring results is required at ~ 7–10 days.

Which MNs are at risk? Among trace elements, those with identified clinical consequences in case of deficiency are copper, selenium, zinc, and iron. These are involved in prolonged neuromuscular weakness (copper), pancytopenia (copper), immune and antioxidant defense, and wound healing [79]. Iron-deficiency anemia confirmed by hepcidin can be treated at end of ICU stay when inflammation abates [80].

### Question 8: How should we personalize monitoring of catabolism and muscle mass?

Acute muscle wasting as signal of catabolism and muscle weakness as associated symptom [81, 82] are ubiquitous in ICU. This results in significant functional disability often persisting for years [83]. Several candidate markers/monitors have been investigated to monitor catabolism to guide practice. The new GLIM malnutrition criteria include objective measure of reduced MM as an essential component of modern malnutrition diagnosis in all patients [84]. Muscle ultrasound is an extensively investigated catabolism measure and used as an outcome measure for interventional trials [66]. Advantages include ease of access, lack of risk/costs, ability to detect necrosis/fasciitis, and longstanding association with physical function in/outside of ICU. Disadvantages include lack of standardization and image acquisition/analysis [85]. A larger coefficient of variation versus other techniques exists and small changes in MM may go undetected. Computed tomography (CT) has much smaller variation coefficient, and MM measurements are standardized [86]. However, it is difficult to envisage protocols allowing for repeated CT MM measurements, given expense, radiation exposure, and logistical/safety issues. That said, new single-slice muscle-specific CT protocols exist, which expose patients to less radiation than chest X-ray and are quick to perform (personal communication-P. Wischmeyer).

Body composition via BIA was long considered unreliable in ICU due to fluid influence on measured components. However, studies using multi-frequency devices show while body composition itself is less exact than DXA, phase angle values and its change over time provide valuable data on cell viability/protein metabolism [87] and LM estimated by BIA predicts outcome [88] as described in “Phase Angle Project” [89].

Biochemical signatures of catabolism are being examined [90]. While metabolomics offer significant granularity and personalization, cost/specialist nature of analysis/interpretation precludes generalizability. The urea-to-creatinine ratio (UCR) is used in physiologic studies and is routinely clinically collected. *UCR is shown to differentiate patients with persistent critical illness, PICS, and post-operative muscle wasting *[93–95]*.* Prospective studies guiding anticatabolic therapies are needed to understand clinical effectiveness of UCR.

### Question 9: How should we personalize use of specialized anabolic nutrients?

Data show protein delivery in ICU via EN is sub-optimal, often below WHO recommendations for protein in healthy populations. This may negatively impact attenuating muscle loss, although timing of ICU-induced catabolism becoming feeding-responsive remains unclear. Thus, exploring single nutrients to stimulate MPS, reduce muscle protein breakdown (MPB), or both are promising. Nutrients frequently used in athletes are interesting in ICU due to ergogenic nature and include leucine, β-hydroxy-β-methylbutyrate (HMB), and creatine. Leucine is an essential AA responsible for initiating anabolic pathways by stimulating mammalian target of rapamycin (mTOR) and acting as substrate for MPS [91]. Only one ICU study has been undertaken as feasibility study and no conclusions on MBP/MPS were drawn [92]. However, meta-analysis shows leucine may improve MM in sarcopenic, elderly persons [93], with potential ICU relevance HMB is a leucine metabolite and stimulates MPS/inhibits MPB [91, 94], making this a widely studied supplement in trained/untrained athletes [95]. A recent systematic review reported improved MM and strength in various clinical populations (not ICU) at risk of muscle wasting [96]. Two recent ICU studies reported no difference in muscle loss, whether measured by ultrasound or CT [87, 97], but its possible duration of intervention was too short for benefit [98, 99]. Creatine’s mechanism increases phosphocreatine within cell and thus increases ATP production essential for MPS. It may provide most benefit to those with lower creatine levels, including potentially critically ill. While no ICU, studies exist, a Cochrane review found short/intermediate-term creatine supplementation improved strength and LM in muscular dystrophies [100].

### Question 10: How to personalize recovery of physical function post-ICU-personalized nutrition, exercise, and anabolic agents?

**Fig. 3:**
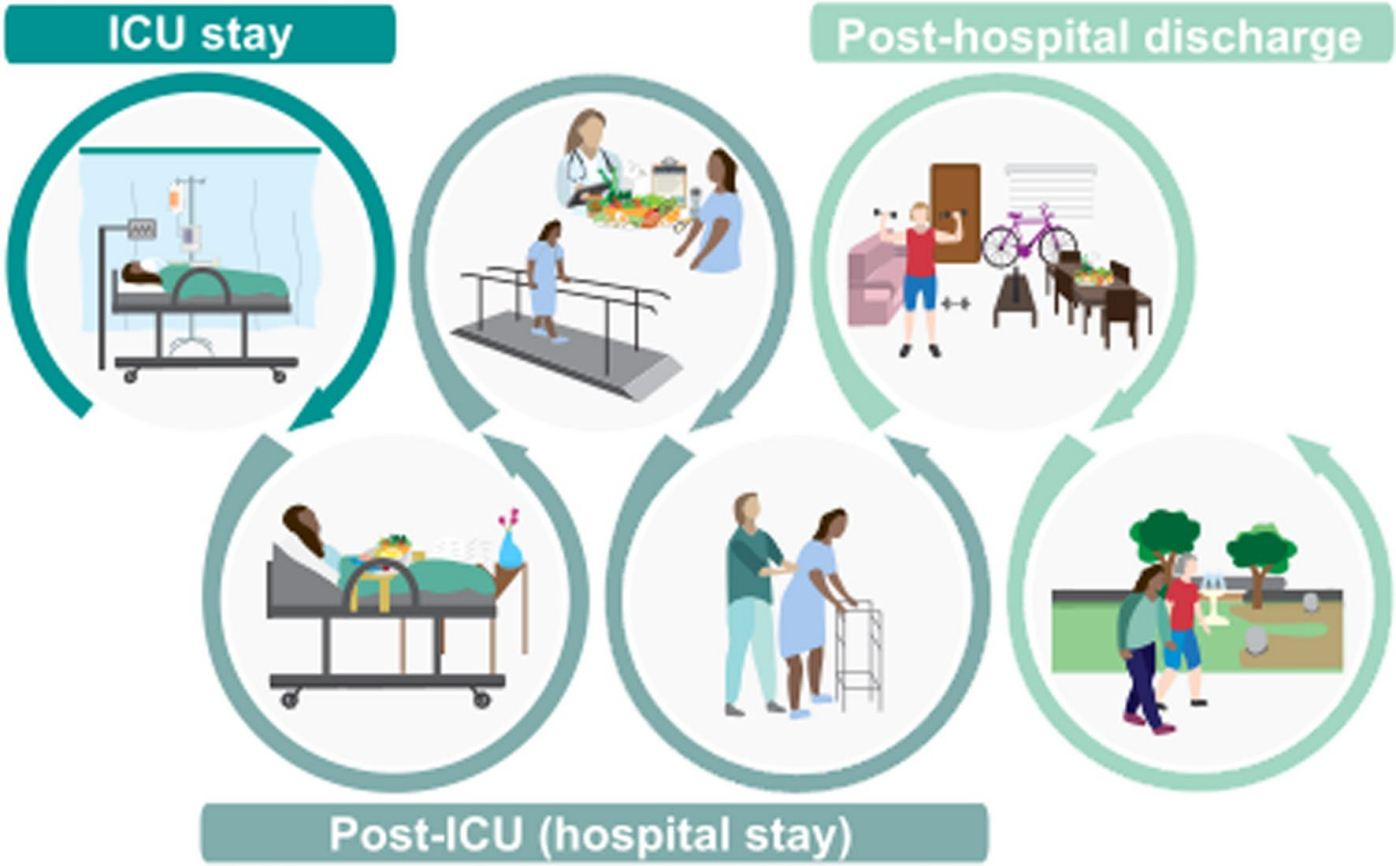
Patient journey from critical care to post-hospital discharge

ICU Survivors frequently suffer significant prolonged physical disability, especially when on ventilator for > 48 h or with significant MOF [101], thus personalized nutrition and exercise across entire ICU patient journey is critical [102] (Fig. 3). “ICU Survivorship” is described as the current “defining challenge in ICU”[103], and existing standards of post-ICU nutrition/rehabilitation care fail to address these disabilities successfully [102]. In addition to catabolic effects of ICU and often prolonged inadequate ICU nutrition delivery, a majority (~ 95%) of patients exhibit severe testosterone deficiency early in ICU [104]. Persistent hypotestosteronemia (Low-T) in acute illness may impair recovery/rehabilitation [104]. Low-T levels correlate with disease severity, ventilator time, ICU LOS, and survival [104, 105]. Benefits of testosterone/testosterone-analogues combined with exercise on clinical outcome/physical function have been demonstrated in range of illnesses [106–108]. In severe burns, multiple trials show oxandrolone (OX) benefits [109], and it is a common standard of care in burn centers worldwide [105]. Meta-analysis showed OX has significant benefits in severe burns, including reduced weight loss, increased LM, improved donor-site healing, and reduced LOS without increase in infection, hyperglycemia, or liver dysfunction [110]. Previous concerns for association of testosterone with cardiovascular/stroke-related events are dispelled by two large studies [111, 112] showing subjects with low-T levels have significant reduction in all-cause cardiovascular events/stroke risk with testosterone compared to untreated [111]. Low-T levels persist into post-ICU period, with 96% T-deficient post-ICU [113]. Research is urgently needed as no current studies exist for multi-modal interventions with testosterone agents in non-burn ICU/post-ICU settings.

Unfortunately, poor nutrition ICU delivery worsens in post-ICU settings. A structured nutrition delivery strategy is optimal for improving this, as described in recent review on ICU/post-ICU nutrition [31]. Another excellent algorithm was described in hospitalized patients at high nutrition risk [114]. The structured algorithm led to significant reductions in mortality and complications at 30 days, and significant improvement in recovery/Functional independence (*p* < 0.006) and EQ-5D QoL at 30 d (*p* = 0.018).

Finally, personalized, exercise programs are becoming key research endeavors and may become critical interventions in ICU recovery. Given unsatisfying results from existing ICU-rehabilitation trials using one-size-fits-all approach, exercise/rehabilitation programs guided by personalized cardiopulmonary exercise testing (CPET) [115] may be key to future ICU rehabilitation. Studies such as the NIH-funded REMM-HIIT (ClinicalTrials.Gov:NCT05218083) utilizing VO2peak heartrate-guided exercise targets are one opportunity for personalized exercise programs to target exercise intensity. These unique CPET-guided heartrate targets allow personalization of home-exercise training guided by mobile technology, as used in REMM-HIIT. Like nutrition, we need to personalize exercise delivery to ICU survivors using wealth of new technologies available from elite athletic world.

## Conclusions

**Fig. 4:**
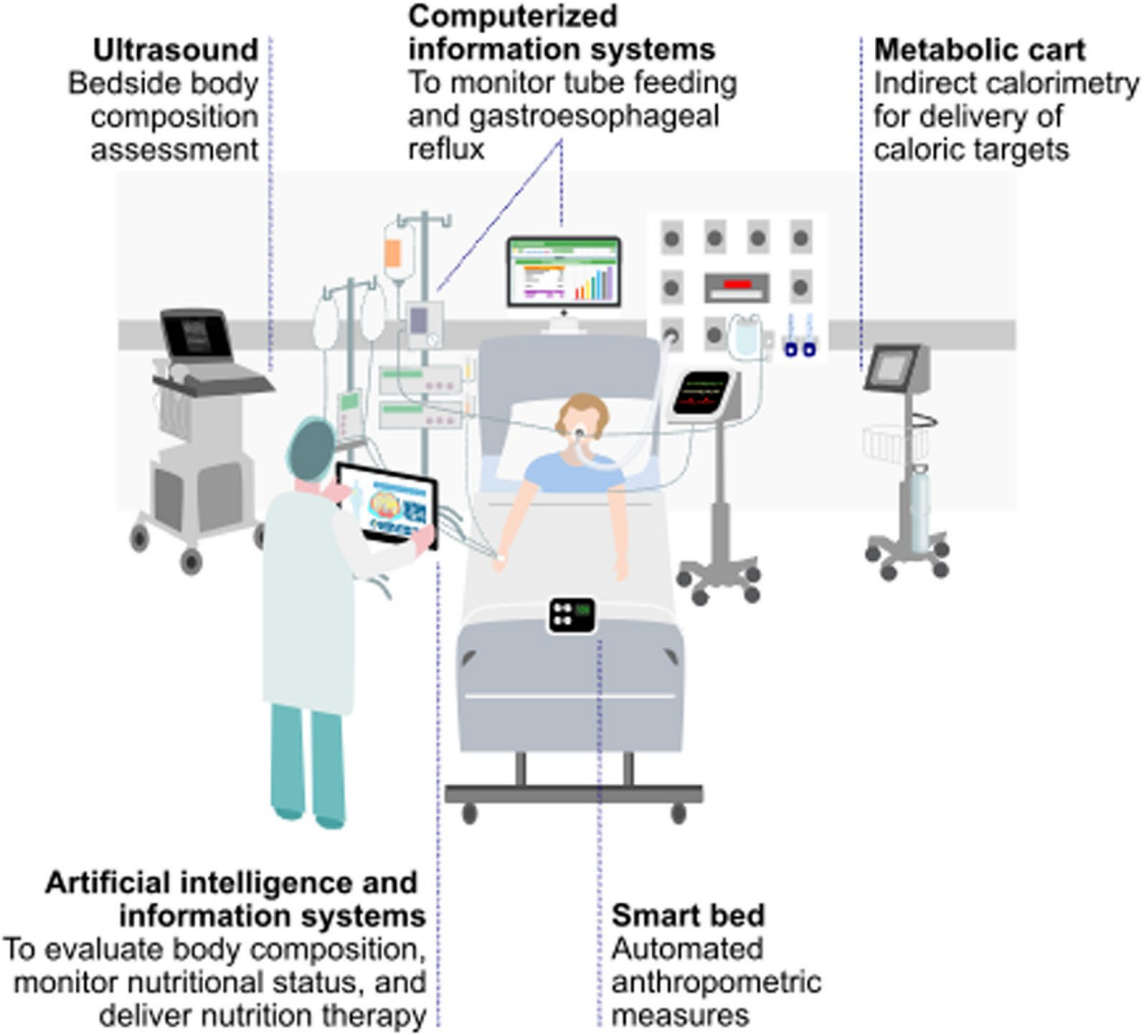
Personalized nutrition care during intensive care unit stay

The importance of personalized ICU nutrition cannot be overstated. We have reached a period in ICU nutrition where we can begin to*”make measurable what has not been so".* We hoped new technologies, such as modern IC devices and unified nutrition platforms (Fig. 4), will ultimately lead to improved clinical/long-term functional outcomes. Most importantly, an urgent need exists to perform trials examining these devices and technologies to determine how to best personalize ICU nutrition to improve outcomes across entire ICU patient journey (Fig. 3). Further, we must develop new markers/technologies, such as markers of when patients can tolerate increased protein/calorie delivery and substrate utilization measures. This is now more needed than ever as critical illness, exemplified by COVID-19 pandemic, poses ever-growing healthcare challenges to improve ICU survival and promote meaningful recovery post-ICU [116].

## Data Availability

Data sharing is not applicable to this article as no datasets were generated or analyzed during the current study.

## References

[1] Wischmeyer PE (2021). Editorial: objective measurement of nutrition and metabolism in the ICU: the future of personalized metabolic therapy. Curr Opin Crit Care.

[2] Singer P, Blaser AR, Berger MM, Alhazzani W, Calder PC, Casaer MP, Hiesmayr M, Mayer K, Montejo JC, Pichard C (2019). ESPEN guideline on clinical nutrition in the intensive care unit. Clin Nutr.

[3] Weimann A, Braga M, Carli F, Higashiguchi T, Hubner M, Klek S, Laviano A, Ljungqvist O, Lobo DN, Martindale R (2017). ESPEN guideline: clinical nutrition in surgery. Clin Nutr.

[4] McClave SA, Taylor BE, Martindale RG, Warren MM, Johnson DR, Braunschweig C, McCarthy MS, Davanos E, Rice TW, Cresci GA (2016). Guidelines for the provision and assessment of nutrition support therapy in the adult critically Ill patient: society of critical care medicine (SCCM) and American society for parenteral and enteral nutrition (ASPEN). JPEN J Parenter Enteral Nutr.

[5] Elke G, Hartl WH, Kreymann KG, Adolph M, Felbinger TW, Graf T, de Heer G, Heller AR, Kampa U, Mayer K (2019). Clinical nutrition in critical care medicine—guideline of the German society for nutritional medicine (DGEM). Clin Nutr ESPEN.

[6] Reintam Blaser A, Starkopf J, Alhazzani W, Berger MM, Casaer MP, Deane AM, Fruhwald S, Hiesmayr M, Ichai C, Jakob SM (2017). Early enteral nutrition in critically ill patients: ESICM clinical practice guidelines. Intensive Care Med.

[7] McClave SA, Lowen CC, Martindale RG (2018). The 2016 ESPEN Arvid Wretlind lecture: the gut in stress. Clin Nutr.

[8] Wischmeyer PE (2020). Enteral nutrition can be given to patients on vasopressors. Crit Care Med.

[9] Fennerty MB (2002). Pathophysiology of the upper gastrointestinal tract in the critically ill patient: rationale for the therapeutic benefits of acid suppression. Crit Care Med.

[10] Krezalek MA, DeFazio J, Zaborina O, Zaborin A, Alverdy JC (2016). The shift of an intestinal "Microbiome" to a "Pathobiome" governs the course and outcome of sepsis following surgical injury. Shock.

[11] McDonald D, Ackermann G, Khailova L, Baird C, Heyland D, Kozar R, Lemieux M, Derenski K, King J, Vis-Kampen C et al: Extreme Dysbiosis of the Microbiome in Critical Illness. mSphere 2016, 1(4). 10.1128/mSphere.00199-16 PMC5007431 27602409

[12] Tian F, Heighes PT, Allingstrup MJ, Doig GS (2018). Early enteral nutrition provided within 24 hours of ICU admission: a meta-analysis of randomized controlled trials. Crit Care Med.

[13] Zhang H, Wang Y, Sun S, Huang X, Tu G, Wang J, Lin Y, Xia H, Yuan Y, Yao S (2019). Early enteral nutrition versus delayed enteral nutrition in patients with gastrointestinal bleeding: a PRISMA-compliant meta-analysis. Medicine (Baltimore).

[14] Haines K, Parker V, Ohnuma T, Krishnamoorthy V, Raghunathan K, Sulo S, Kerr KW, Besecker BY, Cassady BA, Wischmeyer PE (2022). Role of early enteral nutrition in mechanically ventilated COVID-19 patients. Crit Care Explor.

[15] Reignier J, Plantefeve G, Mira JP, Argaud L, Asfar P, Aissaoui N, Badie J, Botoc NV, Brisard L, Bui HN et al: Low versus standard calorie and protein feeding in ventilated adults with shock: a randomised, controlled, multicentre, open-label, parallel-group trial (NUTRIREA-3). Lancet Respir Med 2023. 10.1016/S2213-2600(23)00092-9 36958363

[16] De Waele E, Jonckheer J, Wischmeyer PE (2021). Indirect calorimetry in critical illness: a new standard of care?. Curr Opin Crit Care.

[17] Zusman O, Theilla M, Cohen J, Kagan I, Bendavid I, Singer P (2016). Resting energy expenditure, calorie and protein consumption in critically ill patients: a retrospective cohort study. Crit Care.

[18] De Waele E, Opsomer T, Honoré PM, Diltoer M, Mattens S, Huyghens L, Spapen H (2015). Measured versus calculated resting energy expenditure in critically ill adult patients. Do mathematics match the gold standard?. Minerva Anestesiol.

[19] Oshima T, Berger MM, De Waele E, Guttormsen AB, Heidegger CP, Hiesmayr M, Singer P, Wernerman J, Pichard C (2017). Indirect calorimetry in nutritional therapy. A position paper by the ICALIC study group. Clin Nutr.

[20] Niederer LE, Miller H, Haines KL, Molinger J, Whittle J, MacLeod DB, McClave SA, Wischmeyer PE (2021). Prolonged progressive hypermetabolism during COVID-19 hospitalization undetected by common predictive energy equations. Clin Nutr ESPEN.

[21] Whittle J, Molinger J, MacLeod D, Haines K, Wischmeyer PE (2020). Persistent hypermetabolism and longitudinal energy expenditure in critically ill patients with COVID-19. Crit Care.

[22] McClave SA, Martindale RG, Vanek VW, McCarthy M, Roberts P, Taylor B, Ochoa JB, Napolitano L, Cresci G (2009). Guidelines for the provision and assessment of nutrition support therapy in the adult critically Ill patient: society of critical care medicine (SCCM) and American society for parenteral and enteral nutrition (ASPEN). JPEN J Parenter Enteral Nutr.

[23] Tatucu-Babet OA, Fetterplace K, Lambell K, Miller E, Deane AM, Ridley EJ (2020). Is energy delivery guided by indirect calorimetry associated with improved clinical outcomes in critically Ill patients? A systematic review and meta-analysis. Nutr Metab Insights.

[24] Pertzov B, Bar-Yoseph H, Menndel Y, Bendavid I, Kagan I, Glass YD, Singer P (2022). The effect of indirect calorimetry guided isocaloric nutrition on mortality in critically ill patients-a systematic review and meta-analysis. Eur J Clin Nutr.

[25] Duan JY, Zheng WH, Zhou H, Xu Y, Huang HB (2021). Energy delivery guided by indirect calorimetry in critically ill patients: a systematic review and meta-analysis. Crit Care.

[26] Moonen H, Beckers KJH, van Zanten ARH (2021). Energy expenditure and indirect calorimetry in critical illness and convalescence: current evidence and practical considerations. J Intensive Care.

[27] Wischmeyer PE, Molinger J, Haines K (2021). Point-counterpoint: indirect calorimetry is essential for optimal nutrition therapy in the intensive care unit. Nutr Clin Pract.

[28] Oshima T, Delsoglio M, Dupertuis YM, Singer P, De Waele E, Veraar C, Heidegger CP, Wernermann J, Wischmeyer PE, Berger MM (2020). The clinical evaluation of the new indirect calorimeter developed by the ICALIC project. Clin Nutr.

[29] Oshima T, Delsoglio M, Dupertuis YM, Singer P, De Waele E, Veraar C, Heidegger C-P, Wernermann J, Wischmeyer PE, Berger MM (2020). The clinical evaluation of the new indirect calorimeter developed by the ICALIC project. Clin Nutrit.

[30] Jonckheer J, Spapen H, Malbrain M, Oschima T, De Waele E (2020). Energy expenditure and caloric targets during continuous renal replacement therapy under regional citrate anticoagulation: A viewpoint. Clin Nutr.

[31] van Zanten ARH, De Waele E, Wischmeyer PE (2019). Nutrition therapy and critical illness: practical guidance for the ICU, post-ICU, and long-term convalescence phases. Crit Care.

[32] Puthucheary ZA, Rawal J, McPhail M, Connolly B, Ratnayake G, Chan P, Hopkinson NS, Phadke R, Dew T, Sidhu PS (2013). Acute skeletal muscle wasting in critical illness. JAMA.

[33] Morton RW, Traylor DA, Weijs PJM, Phillips SM (2018). Defining anabolic resistance: implications for delivery of clinical care nutrition. Curr Opin Crit Care.

[34] Weijs PJ, Looijaard WG, Beishuizen A, Girbes AR, Oudemans-van Straaten HM (2014). Early high protein intake is associated with low mortality and energy overfeeding with high mortality in non-septic mechanically ventilated critically ill patients. Crit Care.

[35] Ishibashi N, Plank LD, Sando K, Hill GL (1998). Optimal protein requirements during the first 2 weeks after the onset of critical illness. Crit Care Med.

[36] Lee ZY, Yap CSL, Hasan MS, Engkasan JP, Barakatun-Nisak MY, Day AG, Patel JJ, Heyland DK (2021). The effect of higher versus lower protein delivery in critically ill patients: a systematic review and meta-analysis of randomized controlled trials. Crit Care.

[37] Heyland DK, Patel J, Compher C, Rice TW, Bear DE, Lee ZY, González VC, O'Reilly K, Regala R, Wedemire C (2023). The effect of higher protein dosing in critically ill patients with high nutritional risk (EFFORT Protein): an international, multicentre, pragmatic, registry-based randomised trial. Lancet.

[38] Chapple LS, Kouw IWK, Summers MJ, Weinel LM, Gluck S, Raith E, Slobodian P, Soenen S, Deane AM, van Loon LJC (2022). Muscle protein synthesis after protein administration in critical illness. Am J Respir Crit Care Med.

[39] Casaer MP, Mesotten D, Hermans G, Wouters PJ, Schetz M, Meyfroidt G, Van Cromphaut S, Ingels C, Meersseman P, Muller J (2011). Early versus late parenteral nutrition in critically ill adults. N Engl J Med.

[40] Allingstrup MJ, Kondrup J, Wiis J, Claudius C, Pedersen UG, Hein-Rasmussen R, Bjerregaard MR, Steensen M, Jensen TH, Lange T (2017). Early goal-directed nutrition versus standard of care in adult intensive care patients: the single-centre, randomised, outcome assessor-blinded EAT-ICU trial. Intensive Care Med.

[41] Koekkoek W, van Setten CHC, Olthof LE, Kars J, van Zanten ARH (2019). Timing of PROTein INtake and clinical outcomes of adult critically ill patients on prolonged mechanical VENTilation: The PROTINVENT retrospective study. Clin Nutr.

[42] van Gassel RJJ, Baggerman MR, van de Poll MCG (2020). Metabolic aspects of muscle wasting during critical illness. Curr Opin Clin Nutr Metab Care.

[43] Heyland D, Muscedere J, Wischmeyer PE, Cook D, Jones G, Albert M, Elke G, Berger MM, Day AG (2013). Canadian critical care trials G: a randomized trial of glutamine and antioxidants in critically ill patients. N Engl J Med.

[44] Moonen H, Van Zanten ARH (2021). Bioelectric impedance analysis for body composition measurement and other potential clinical applications in critical illness. Curr Opin Crit Care.

[45] Gallagher D, Heymsfield SB, Heo M, Jebb SA, Murgatroyd PR, Sakamoto Y (2000). Healthy percentage body fat ranges: an approach for developing guidelines based on body mass index. Am J Clin Nutr.

[46] Kim TJ, Park SH, Jeong HB, Ha EJ, Cho WS, Kang HS, Kim JE, Ko SB: Optimizing Nitrogen Balance Is Associated with Better Outcomes in Neurocritically Ill Patients. Nutrients 2020, 12(10). 10.3390/nu12103137 PMC7602201 33066539

[47] Harimawan AIW, Dewi NMRP, Samsarga GW (2021). Nitrogen balance for estimating protein requirements in critically ill patients: a literature review. Indonesia J Biomed Sci.

[48] Heidegger CP, Berger MM, Graf S, Zingg W, Darmon P, Costanza MC, Thibault R, Pichard C (2013). Optimisation of energy provision with supplemental parenteral nutrition in critically ill patients: a randomised controlled clinical trial. Lancet.

[49] Doig GS, Simpson F, Sweetman EA, Finfer SR, Cooper DJ, Heighes PT, Davies AR, O'Leary M, Solano T, Peake S (2013). Early parenteral nutrition in critically ill patients with short-term relative contraindications to early enteral nutrition: a randomized controlled trial. JAMA.

[50] Reignier J, Boisrame-Helms J, Brisard L, Lascarrou JB, Ait Hssain A, Anguel N, Argaud L, Asehnoune K, Asfar P, Bellec F (2018). Enteral versus parenteral early nutrition in ventilated adults with shock: a randomised, controlled, multicentre, open-label, parallel-group study (NUTRIREA-2). Lancet.

[51] Compher C, Bingham AL, McCall M, Patel J, Rice TW, Braunschweig C, McKeever L (2022). Guidelines for the provision of nutrition support therapy in the adult critically ill patient: the American society for parenteral and enteral nutrition. JPEN J Parenter Enteral Nutr.

[52] Harvey SE, Parrott F, Harrison DA, Bear DE, Segaran E, Beale R, Bellingan G, Leonard R, Mythen MG, Rowan KM (2014). Trial of the route of early nutritional support in critically ill adults. N Engl J Med.

[53] Hill A, Heyland DK, Ortiz Reyes LA, Laaf E, Wendt S, Elke G, Stoppe C (2021). Combination of enteral and parenteral nutrition in the acute phase of critical illness: an updated systematic review and meta-analysis. JPEN J Parenter Enteral Nutr.

[54] Wischmeyer PE, Hasselmann M, Kummerlen C, Kozar R, Kutsogiannis DJ, Karvellas CJ, Besecker B, Evans DK, Preiser JC, Gramlich L (2017). A randomized trial of supplemental parenteral nutrition in underweight and overweight critically ill patients: the TOP-UP pilot trial. Crit Care.

[55] Gao X, Liu Y, Zhang L, Zhou D, Tian F, Gao T, Tian H, Hu H, Gong F, Guo D (2022). Effect of early vs late supplemental parenteral nutrition in patients undergoing abdominal surgery: a randomized clinical trial. JAMA Surg.

[56] Hill A, Heyland DK, Ortiz Reyes LA, Laaf E, Wendt S, Elke G, Stoppe C (2022). Combination of enteral and parenteral nutrition in the acute phase of critical illness: an updated systematic review and meta-analysis. JPEN J Parenter Enteral Nutr.

[57] Alsharif DJ, Alsharif FJ, Aljuraiban GS, Abulmeaty MMA (2020). Effect of supplemental parenteral nutrition versus enteral nutrition alone on clinical outcomes in critically ill adult patients: a systematic review and meta-analysis of randomized controlled trials. Nutrients.

[58] Puthucheary Z, Gunst J (2021). Are periods of feeding and fasting protective during critical illness?. Curr Opin Clin Nutr Metab Care.

[59] Gunst J (2017). Recovery from critical illness-induced organ failure: the role of autophagy. Crit Care.

[60] De Bruyn A, Gunst J, Goossens C, Vander Perre S, Guerra GG, Verbruggen S, Joosten K, Langouche L, Van den Berghe G (2020). Effect of withholding early parenteral nutrition in PICU on ketogenesis as potential mediator of its outcome benefit. Crit Care.

[61] De Bruyn A, Langouche L, Vander Perre S, Gunst J, Van den Berghe G (2021). Impact of withholding early parenteral nutrition in adult critically ill patients on ketogenesis in relation to outcome. Crit Care.

[62] Atherton PJ, Etheridge T, Watt PW, Wilkinson D, Selby A, Rankin D, Smith K, Rennie MJ (2010). Muscle full effect after oral protein: time-dependent concordance and discordance between human muscle protein synthesis and mTORC1 signaling. Am J Clin Nutr.

[63] Allada R, Bass J (2021). Circadian mechanisms in medicine. N Engl J Med.

[64] Van Dyck L, Vanhorebeek I, Wilmer A, Schrijvers A, Derese I, Mebis L, Wouters PJ, Van den Berghe G, Gunst J, Casaer MP (2020). Towards a fasting-mimicking diet for critically ill patients: the pilot randomized crossover ICU-FM-1 study. Crit Care.

[65] Van Dyck L, Casaer MP (2019). Intermittent or continuous feeding: any difference during the first week?. Curr Opin Crit Care.

[66] McNelly AS, Bear DE, Connolly BA, Arbane G, Allum L, Tarbhai A, Cooper JA, Hopkins PA, Wise MP, Brealey D (2020). Effect of intermittent or continuous feed on muscle wasting in critical illness: a phase 2 clinical trial. Chest.

[67] Van Dyck L, Gunst J, Casaer MP, Peeters B, Derese I, Wouters PJ, de Zegher F, Vanhorebeek I, Van den Berghe G (2020). The clinical potential of GDF15 as a "ready-to-feed indicator" for critically ill adults. Crit Care.

[68] Alberda C, Gramlich L, Jones N, Jeejeebhoy K, Day A, Dhaliwal R, Heyland D (2009). The relationship between nutritional intake and clinical outcomes in critically ill patients: results of an international multicenter observational study. Intensive Care Med.

[69] Berger M, Revelly J, Wasserfallen J, Schmid A, Bouvry S, Cayeux M, Musset M, Maravic P, Chiolero R (2006). Impact of a computerized information system on quality of nutritional support in the ICU. Nutrition.

[70] Singer P, Lia L (2019). Technology innovations in delivering accurate nutrition. ICU Management Practice.

[71] Berger M, Reintam-Blaser A, Calder P, Casaer M, Hiesmayr M, Mayer K, Montejo J, Pichard C, Preiser J, van Zanten A (2019). Monitoring nutrition in the ICU. Clin Nutr.

[72] Vankrunkelsven W, Gunst J, Amrein K, Bear DE, Berger MM, Christopher KB, Fuhrmann V, Hiesmayr MJ, Hiesmayr MJ, Ichai C et al. Current practice and variability in micronutrient monitoring and administration: results of the vita-trace survey. Critical care 2019, 23.

[73] Berger M, Broman M, Forni L, Ostermann M, De Waele E, Wischmeyer P (2021). Nutrients and micronutrients at risk during renal replacement therapy: a scoping review. Curr Opin Crit Care.

[74] Fah M, Van Althuis LE, Ohnuma T, Winthrop HM, Haines KL, Williams DGA, Krishnamoorthy V, Raghunathan K, Wischmeyer PE (2022). Micronutrient deficiencies in critically ill patients receiving continuous renal replacement therapy. Clin Nutr ESPEN.

[75] Berger MM, Shenkin A, Schweinlin A, Amrein K, Augsburger M, Biesalski HK, Bischoff SC, Casaer MP, Gundogan K, Lepp HL (2022). ESPEN micronutrient guideline. Clin Nutr.

[76] Berger M, Pantet O, Schneider A, Ben-Hamouda N (2019). Micronutrient deficiencies in medical and surgical inpatients. J Clin Med.

[77] Duncan A, Talwar D, McMillan D, Stefanowicz F, O'Reilly D (2012). Quantitative data on the magnitude of the systemic inflammatory response and its effect on micronutrient status based on plasma measurements. Am J Clin Nutr.

[78] Altarelli M, Ben-Hamouda N, Schneider A, Berger M (2019). Copper deficiency—causes, manifestations, and treatment. Nutr Clin Pract.

[79] Berger MM, Ben-Hamouda N (2020). Trace element and vitamin deficiency: quantum medicine or essential prescription?. Curr Opin Crit Care.

[80] Lasocki S, Asfar P, Jaber S, Ferrandiere M, Kerforne T, Asehnoune K, Montravers P, Seguin P, Peoc'h K, Gergaud S (2021). Impact of treating iron deficiency, diagnosed according to hepcidin quantification, on outcomes after a prolonged ICU stay compared to standard care: a multicenter, randomized, single-blinded trial. Crit Care.

[81] Puthucheary ZA, McNelly AS, Rawal J, Connolly B, Sidhu PS, Rowlerson A, Moxham J, Harridge SD, Hart N, Montgomery HE (2017). Rectus femoris cross-sectional area and muscle layer thickness: comparative markers of muscle wasting and weakness. Am J Respir Crit Care Med.

[82] Puthucheary ZA, Rawal J, McPhail M, Connolly B, Ratnayake G, Chan P, Hopkinson NS, Padhke R, Dew T, Sidhu PS (2013). Acute skeletal muscle wasting in critical illness. JAMA.

[83] Herridge MS, Tansey CM, Matte A, Tomlinson G, Diaz-Granados N, Cooper A, Guest CB, Mazer CD, Mehta S, Stewart TE (2011). Functional disability 5 years after acute respiratory distress syndrome. N Engl J Med.

[84] Cederholm T, Jensen GL, Correia M, Gonzalez MC, Fukushima R, Higashiguchi T, Baptista G, Barazzoni R, Blaauw R, Coats A (2019). GLIM criteria for the diagnosis of malnutrition—a consensus report from the global clinical nutrition community. Clin Nutr.

[85] Mourtzakis M, Parry S, Connolly B, Puthucheary Z (2017). Skeletal muscle ultrasound in critical care: a tool in need of translation. Ann Am Thorac Soc.

[86] Bear DE, MacGowan L, Elstad M, Puthucheary Z, Connolly B, Wright R, Hart N, Harridge S, Whelan K, Barrett NA (2021). Relationship between skeletal muscle area and density and clinical outcome in adults receiving venovenous extracorporeal membrane oxygenation. Crit Care Med.

[87] Viana M, Becce F, Pantet O, Schmidt S, Bagnoud G, Thaden J, Ten Have G, Engelen M, Voidey A, Deutz N (2021). Impact of β−hydroxy-β−methylbutyrate (HMB) on muscle loss and protein metabolism in critically ill patients: A RCT. Clin Nutr.

[88] Thibault R, Makhlouf A, Mulliez A, Cristina Gonzalez M, Kekstas G, Kozjek N, Preiser J, Ceniceros I, Rozalen I, Dadet S (2016). Fat-free mass at admission predicts 28-day mortality in intensive care unit patients: the international prospective observational study phase angle project. Intensive Care Med.

[89] Ko SJ, Cho J, Choi SM, Park YS, Lee CH, Lee SM, Yoo CG, Kim YW, Lee J (2021). Phase angle and frailty are important prognostic factors in critically Ill medical patients: a prospective cohort study. J Nutr Health Aging.

[90] Page A, Flower L, Prowle J, Puthucheary Z (2021). Novel methods to identify and measure catabolism. Curr Opin Crit Care.

[91] Wilkinson DJ, Hossain T, Hill DS, Phillips BE, Crossland H, Williams J, Loughna P, Churchward-Venne TA, Breen L, Phillips SM (2013). Effects of leucine and its metabolite beta-hydroxy-beta-methylbutyrate on human skeletal muscle protein metabolism. J Physiol.

[92] Wandrag L, Brett SJ, Frost GS, To M, Loubo EA, Jackson NC, Umpleby AM, Bountziouka V, Hickson M (2019). Leucine-enriched essential amino acid supplementation in mechanically ventilated trauma patients: a feasibility study. Trials.

[93] Gielen E, Beckwée D, Delaere A, De Breucker S, Vandewoude M, Bautmans I: Nutritional interventions to improve muscle mass, muscle strength, and physical performance in older people: an umbrella review of systematic reviews and meta-analyses. Nutrition reviews 2020. 10.1093/nutrit/nuaa011 32483625

[94] Wilkinson DJ, Hossain T, Limb MC, Phillips BE, Lund J, Williams JP, Brook MS, Cegielski J, Philp A, Ashcroft S (2018). Impact of the calcium form of β-hydroxy-β-methylbutyrate upon human skeletal muscle protein metabolism. Clin Nutr.

[95] Wilson JM, Fitschen PJ, Campbell B, Wilson GJ, Zanchi N, Taylor L, Wilborn C, Kalman DS, Stout JR, Hoffman JR (2013). International Society of Sports Nutrition Position Stand: beta-hydroxy-beta-methylbutyrate (HMB). J Int Soc Sports Nutr.

[96] Bear DE, Langan A, Dimidi E, Wandrag L, Harridge SDR, Hart N, Connolly B, Whelan K (2019). Beta-Hydroxy-beta-methylbutyrate and its impact on skeletal muscle mass and physical function in clinical practice: a systematic review and meta-analysis. Am J Clin Nutr.

[97] Nakamura K, Kihata A, Naraba H, Kanda N, Takahashi Y, Sonoo T, Hashimoto H, Morimura N (2020). β-Hydroxy-β-methylbutyrate, arginine, and glutamine complex on muscle volume loss in critically Ill patients: a randomized control trial. JPEN J Parenter Enteral Nutr.

[98] Bear DE, Puthucheary ZA (2019). Designing nutrition-based interventional trials for the future: addressing the known knowns. Crit Care.

[99] Bear DE, Wandrag L, Merriweather JL, Connolly B, Hart N, Grocott MPW (2017). Enhanced recovery after critical illness programme group i: the role of nutritional support in the physical and functional recovery of critically ill patients: a narrative review. Crit Care.

[100] Kley RA, Tarnopolsky MA, Vorgerd M (2013). Creatine for treating muscle disorders. Cochrane Database Syst Rev.

[101] Herridge MS, Azoulay É (2023). Outcomes after critical illness. N Engl J Med.

[102] Molinger J, Pastva AM, Whittle J, Wischmeyer PE (2020). Novel approaches to metabolic assessment and structured exercise to promote recovery in ICU survivors. Curr Opin Crit Care.

[103] Iwashyna TJ (2010). Survivorship will be the defining challenge of critical care in the 21st century. Ann Intern Med.

[104] Almoosa KF, Gupta A, Pedroza C, Watts NB (2014). low testosterone levels are frequent in patients with acute respiratory failure and are associated with poor outcomes. Endocr Pract.

[105] Wischmeyer PE, Suman OE, Kozar R, Wolf SE, Molinger J, Pastva AM (2020). Role of anabolic testosterone agents and structured exercise to promote recovery in ICU survivors. Curr Opin Crit Care.

[106] Toma M, McAlister FA, Coglianese EE, Vidi V, Vasaiwala S, Bakal JA, Armstrong PW, Ezekowitz JA (2012). Testosterone supplementation in heart failure: a meta-analysis. Circ Heart Fail.

[107] Sardar P, Jha A, Roy D, Majumdar U, Guha P, Roy S, Banerjee R, Banerjee AK, Bandyopadhyay D (2010). Therapeutic effects of nandrolone and testosterone in adult male HIV patients with AIDS wasting syndrome (AWS): a randomized, double-blind, placebo-controlled trial. HIV Clin Trials.

[108] Schols AM, Soeters PB, Mostert R, Pluymers RJ, Wouters EF (1995). Physiologic effects of nutritional support and anabolic steroids in patients with chronic obstructive pulmonary disease. A placebo-controlled randomized trial. Am J Respir Crit Care Med.

[109] Stanojcic M, Finnerty CC, Jeschke MG (2016). Anabolic and anticatabolic agents in critical care. Curr Opin Crit Care.

[110] Li H, Guo Y, Yang Z, Roy M, Guo Q (2016). The efficacy and safety of oxandrolone treatment for patients with severe burns: A systematic review and meta-analysis. Burns.

[111] Cheetham TC, An J, Jacobsen SJ, Niu F, Sidney S, Quesenberry CP, VanDenEeden SK (2017). Association of testosterone replacement with cardiovascular outcomes among men with androgen deficiency. JAMA Intern Med.

[112] Anderson JL, May HT, Lappe DL, Bair T, Le V, Carlquist JF, Muhlestein JB (2016). Impact of testosterone replacement therapy on myocardial infarction, stroke, and death in men with low testosterone concentrations in an integrated health care system. Am J Cardiol.

[113] Nierman DM, Mechanick JI (1999). Hypotestosteronemia in chronically critically ill men. Crit Care Med.

[114] Schuetz P, Fehr R, Baechli V, Geiser M, Deiss M, Gomes F, Kutz A, Tribolet P, Bregenzer T, Braun N (2019). Individualised nutritional support in medical inpatients at nutritional risk: a randomised clinical trial. Lancet.

[115] Whittle J, San-Millán I (2021). Objective assessment of metabolism and guidance of ICU rehabilitation with cardiopulmonary exercise testing. Curr Opin Crit Care.

[116] Berlin D, Gulick R, Martinez F (2020). Severe Covid-19. N Engl J Med.

